# Volatile Emissions From Diverse Estuarine Bacteria Share Core Compounds With a Subset of Strain‐Specific, Low Abundance Compounds

**DOI:** 10.1111/1758-2229.70439

**Published:** 2026-10-18

**Authors:** Eve Galen, Kajsa Roslund, Riikka Rinnan, Lasse Riemann

**Affiliations:** ^1^ Center for Volatile Interactions, Department of Biology University of Copenhagen Copenhagen E Denmark; ^2^ Marine Biological Section, Department of Biology University of Copenhagen Helsingør Denmark

**Keywords:** Baltic Sea, biogenic volatile organic compounds (BVOC), headspace analysis, marine heterotrophic bacteria, PTR‐TOF‐MS, volatilome

## Abstract

Biogenic volatile organic compounds (BVOCs) are gases that influence atmospheric chemistry, nutrient cycling and species interactions, yet the contribution of heterotrophic marine bacteria to marine BVOC emissions remains poorly constrained. In addition, the extent to which the volatilome is linked to bacterial phylogeny is unknown. Here, we characterize the volatilome of 16 heterotrophic bacterial strains isolated from Baltic Sea surface water, spanning Alphaproteobacteria, Gammaproteobacteria, Betaproteobacteria, Bacteroidota and Actinomycetes. Headspace BVOCs were quantified under standardized growth conditions using Proton Transfer Reaction Time‐of‐Flight Mass Spectrometry (PTR‐TOF‐MS). A broadly overlapping bacterial volatilome was identified, with compound composition and proportional abundance similar across many strains, irrespective of phylogeny. Namely, most strains shared a set of core compounds with a subset of strain‐specific, low abundance compounds. Acetone accounted for more than 50% of the emissions in most volatilomes. The remaining fraction of emissions was primarily comprised of other low‐molecular‐weight oxygenated compounds. Interestingly, two strains demonstrated strain‐specific emission patterns, significantly diverging from the group in their emission rate and compound composition. Together, these findings suggest that marine heterotrophic bacteria may contribute a broadly conserved collection of BVOCs to the ocean–atmosphere interface, highlighting their role as a widespread source of trace gases in marine ecosystems.

## Graphical abstract

Biogenic volatile organic compounds (BVOCs) emissions from 16 heterotrophic bacterial strains cultivated from Baltic Sea surface waters were quantified using PTR‐ToF‐MS. Strains showed broadly overlapping volatilomes with no clear phylogenetic relationship. Acetone was highly abundant in most strains, along with a suite of core compounds. Two strains showed divergent emission profiles, providing new insight into the structure and controls of marine bacterial volatilomes.

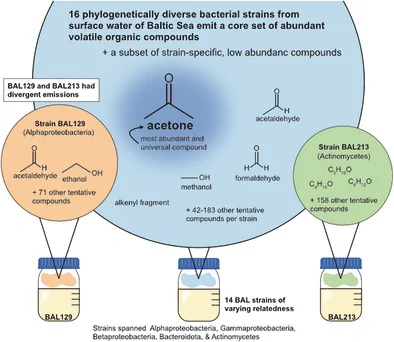

## 1 Introduction

Biogenic volatile organic compounds (BVOCs) are generally characterized by their size (< 300 Da) and high vapour pressure, which enables them to easily diffuse in water and air (Weisskopf et al. 2021). These carbon‐based compounds are produced by a variety of biosynthetic pathways, including primary and secondary metabolism, fermentation, sulphur metabolism, fatty acid biosynthesis and terpene synthesis (Schulz and Dickschat 2007; Weisskopf et al. 2021). Emission of BVOCs to the atmosphere has consequences for carbon cycling as well as regional and global climate. Namely, the oxidation of BVOCs can form secondary organic aerosols, affect the atmosphere's oxidative capacity and impact the lifetime of greenhouse gases (Atkinson and Arey 2003; Hallquist et al. 2009; Fu et al. 2010).

Many studies have reported the production of hydrocarbons and organic sulphur compounds by phytoplankton (Simó 2001; Shaw et al. 2010; Halsey et al. 2017; Davie‐Martin et al. 2020; Rocco et al. 2021; Halsey and Giovannoni 2023), however, less understood is the role of heterotrophic bacteria in the production of these compounds and other BVOCs. Bacteria are responsible for carbon cycling in marine waters (Azam et al. 1983; Azam 1998), however, information about the identity and abundance of the BVOCs they produce is sparse. Recently, bacterial assemblages from Japanese coastal waters were found to emit acetaldehyde, acetone and four sulphur compounds, including dimethyl sulphide (DMS) and methanethiol (MeSH; Omori et al. 2025). A few previous studies have also confirmed the production of acetone (Nemecek‐Marshall et al. 1995, 1999), DMS and MeSH (Sun et al. 2016) by marine heterotrophic bacteria as well as methyl halides (Amachi et al. 2001; Fujimori et al. 2012). No studies have reported on the total BVOC composition, named the volatilome, produced by marine bacteria. The overall bacterial contribution to marine BVOC emissions is, therefore, poorly understood.

Marine bacterial communities harbour populations with distinct metabolisms and divergent resource preferences, assimilating and transforming organic matter, including BVOCs, in diverse manners (Bunse and Pinhassi 2017). Populations may therefore have disparate effects on the cycling of specific compounds. Still, it is unknown to what extent marine bacterial populations emit characteristic BVOCs and whether the volatilome can be linked to community composition dynamics. Two previous studies indicate that microbial BVOC composition is partially structured by phylogeny, with taxonomy explaining 34%–52% of variation in BVOC composition among Actinobacteria (Choudoir et al. 2019) and approximately 12% of bacterial and fungal BVOCs exhibiting a significant phylogenetic signal (Elmassry et al. 2020). However, in both studies, most variation in microbial volatilomes was not attributable to phylogenetic relatedness. Indeed, in other studies bacterial volatilomes were dynamic, with strain‐specific variability strongly influenced by environmental conditions (Cordovez et al. 2015; Bailly and Weisskopf 2017; Fitzgerald et al. 2021). Hence, the coupling between phylogeny and volatilomes appears unclear, particularly for marine bacteria. This information is essential for identifying drivers regulating microbial BVOC emissions and for an ecological understanding of BVOC dynamics. Furthermore, research on marine BVOCs has primarily focused on targeted compounds such as isoprene, DMS and its precursors and select oxygenated BVOCs, like methanol, acetaldehyde and acetone. More than 3500 microbial BVOCs have been directly measured across diverse terrestrial and anthropogenic environments and under laboratory culture conditions (Kemmler et al. 2025). This reflects the vast breadth of microbial emissions, yet few studies have addressed the chemical diversity of marine microbial BVOCs (Krause et al. 2025; Salinas‐García et al. 2025). Studying the volatilome of marine bacteria may lend new information to distinct pathways of bacterial metabolism and insight into the bacterial contribution to pools and fluxes of specific BVOCs in marine waters.

Decoupling community‐level and individual‐level emissions remains a major challenge for in situ BVOC measurements. Currently, community‐averaged measurements likely obscure strain‐specific contributions to BVOC fluxes, masking the unique attributes and traits of different species. For example, single strains may be responsible for disproportionate emission rates or emit unique volatilomes, indicating a distinctive function in the environment or an outsized impact on biogeochemical cycling of BVOCs (Weisskopf et al. 2021). Moreover, most studies do not disentangle BVOC emissions between phytoplankton and heterotrophic bacteria, with only a few studies measuring phytoplankton emissions with and without bacteria present (Moore et al. 2020; Padaki et al. 2025). Overall, only a few studies have investigated BVOC emissions from marine bacteria alone, with all studies either focusing on a single compound or a few, phylogenetically similar strains (Nemecek‐Marshall et al. 1995, 1999; Thiel et al. 2009; Groenhagen et al. 2016; Lawson et al. 2020; Chhalodia et al. 2021). To date, no studies have characterized marine bacterial volatilomes across different phylogeny or used a high‐sensitivity approach optimized for targeting low‐molecular‐weight oxygenated compounds, such as Proton Transfer Reaction Time‐of‐Flight Mass Spectrometry (PTR‐ToF‐MS).

Here, we report the emission rates and volatilomes of 16 diverse bacterial strains isolated from the Baltic Sea. Specifically, we examined the patterns between strain phylogeny and their volatilomes. As nutrient conditions, genomic variation, growth phase and proximity to other species are known to influence the abundance and blend of BVOC emissions, all factors except genomic variation were kept constant (Choudoir et al. 2019). By comparing volatilomes across closely and distantly related strains, we aimed to study whether emission patterns are conserved along evolutionary lineages or if volatilomes reflect strain‐specific adaptations.

## 2 Materials and Methods

### 2.1 Bacterial Strains

Bacterial isolates were originally obtained from surface water samples (3 m) collected at the Landsort Deep station (BY31; 58° 35′ N, 18° 14′ E) in the Baltic Sea Proper between March 2003 and May 2004. Detailed procedures for water sampling, bacterial isolation and 16S rRNA amplicon sequencing are described in Riemann et al. (2008). For the present study, 16 strains were selected from within Alphaproteobacteria, Gammaproteobacteria, Betaproteobacteria, Bacteroidota and Actinomycetota (Table S1). Strains of varying phylogenetic relatedness were chosen for comparison, including a set of identical (in morphology and nucleotide identity) strains from different stocks for the purpose of validating our approach.

### 2.2 Bacterial Cultivation and Growth

Zobell marine medium of salinity 16–17 was prepared for broth and solid applications by mixing 5 g peptone, 1 g yeast extract and 800 mL surface seawater from Øresund, Denmark and 200 mL Milli‐Q water and then autoclaving (Zobell 1941). M9 minimal medium (salinity 16–17) was prepared by adding 1 g NH_4_Cl, 6 g Na_2_HPO_4_·7H_2_O, 3 g KH_2_PO_4_ and 0.5 g NaCl to 1 L of Milli‐Q water, autoclaved and then adding filter sterilized MgSO_4_ and glucose to final concentrations of 2 and 22 mM, respectively (Miller 1972).

Strains were grown on Zobell agar plates. Single colonies were inoculated into Zobell broth and grown at 19°C while shaking (250 rpm) until visible turbidity was achieved. Cells were harvested by centrifugation at 4000 × *g* for 6 min. The pellet was gently resuspended in 1 mL of M9 medium. This washing step was repeated once and each of the triplicate pellets was resuspended in 26 mL of M9 medium in 50 mL Falcon tubes. Medium‐only controls were prepared in parallel.

Cell growth was monitored by optical density (OD_600_) in an Eppendorf BioPhotometer (Hamburg, Germany). After a few days of growth, cells were diluted to an OD of 0.1 in 30 mL of M9 medium in 100 mL Duran bottles. After dilution, OD was recorded every 6–12 h to identify the mid‐exponential phase of growth, at which time point BVOCs were sampled from a parallel set of cultures. Immediately prior to BVOC analysis, aliquots were removed from the culture for OD measurement for growth phase confirmation and cell enumeration (fixed with 1% glutaraldehyde for 10 min at room temperature and stored at −80°C). Cells were enumerated on a FACSCanto II flow cytometer (BD, New Jersey, USA) using SYBR Green I staining (Brussaard et al. 2010) and TrueCount beads (BD) were used to measure the flow rate.

### 2.3 Measurement of BVOCs

Duran bottles (100 mL) containing 30 mL of culture were fitted with modified lids containing an inlet and an outlet port and incubated in a dark thermal chamber at 19°C with shaking (250 rpm). The inlet port was connected to a zero‐air generator (Tisch Environmental, 747‐30, Advanced Ultra‐Pure Air Generator, OH, USA) delivering a continuous BVOC‐free air flow of 4.8 L h^−1^. The outlet was connected to the PTR‐ToF‐MS (1000 ultra, IONICON Analytik, Austria) for real‐time BVOC analysis. Ion counts of several *m/*z of interest (e.g., *m/z* 33, methanol; *m/z* 45, acetaldehyde; *m/z* 59, acetone; *m/z* 69, isoprene) were monitored until a steady‐state signal was reached, defined as a stable ion count for a minimum of 5 min. The time to reach steady‐state varied between samples (30–120 min). Biological replicates were analysed across seven measurement days over a one‐month period. Replicates of each strain were analysed on the same day but distributed throughout the measurement sequence rather than analysed consecutively. Strains from the same phylum/class were measured on different days to avoid systemic grouping of related strains. Medium‐only controls were measured daily under the same conditions as the bacterial cultures.

For measurement with the PTR‐ToF‐MS, hydronium (H_3_O^+^) was used as the reagent ion and measurements were performed from a mass‐to‐charge ratio (*m/z*) 17–239 with a 0.25 Hz measurement frequency. The PTR‐ToF‐MS operational parameters were as follows: drift tube pressure of 2.20 mbar, temperature of 60°C and voltage of 500 V, corresponding to a reduced electric field (*E*/*N*) of 116 Td. Ion source current of 3.0 mA and a H_2_O flow of 6.0 standard cubic centimetres per minute (sccm). Instrument inlet flow was 25 sccm and temperature was 70°C. For mass scale calibration, an internal standard (1,3‐diiodobenzene, *m/z* 203.94) and the H_3_(16)O^+^ (*m/z* 21.02) peaks were used. Transmission curves and instrument calibration were achieved with a standard gas mixture (Air Liquide, Ballerup, Denmark) with known concentrations of 11 BVOCs in pure nitrogen (Table S2). The calibration data were used for compound identification. Compounds missing an authentic standard were tentatively identified using the GLOVOCS database (Yáñez‐Serrano et al. 2021) in combination with a targeted literature review. Compound assignment was carried out for strain‐specific *m/z* and the 10 most abundant *m/z* for each strain.

### 2.4 Post‐Processing and BVOC Emission Rate Calculation

Raw data from the PTR‐ToF‐MS were processed using PTRwid (version 3, 2018; Holzinger 2015), which performs automated mass axis calibration and peak detection and constructs a unified mass list across samples. Peak identification and subsequent unified mass list were manually confirmed by cross‐comparison with raw spectra using PTR‐MS Viewer v3.4.4.13 (IONICON Analytik, Austria). Ion source‐derived species (e.g., O_2_^+^, H_3_O^+^, NO_2_^+^) and interfering signals such as hydrate clusters and other artefacts were excluded from the final mass list.

Data processing, visualization, phylogenetic analysis and statistical analysis were performed using R version 4.3.3. Raw ion counts were converted to volume mixing ratios (in parts per billion by volume (ppbv) via Equation 1).

(1) $$C=\frac{i\left(MH^{+}\right)}{i\left(H_{3}O^{+}\right)}\frac{\mathrm{Tr}\left(H_{3}O^{+}\right)}{\mathrm{Tr}\left(MH^{+}\right)}\frac{1}{\mathrm{kt}}\frac{\mathrm{RT}}{pN_{A}}$$

where *i*(MH^+^) is the ion current (in ion counts per second) measured for the protonated sample molecule and i(H_3_O^+^) for the precursor ion. *N*_*A*_ is the Avogadro's constant, *R* is the gas constant and *p* and *T* are the drift tube pressure and temperature, respectively, *k* is the rate coefficient and *t* is the reaction time. Transmission factors (*Tr*) for different sample ions and the precursor ion were measured with the calibration gas mixture, to account for mass‐dependence of the proton‐transfer‐reaction rate. Volume mixing ratio (*C*) is the partial volume of a compound in a mixture divided by the total volume, expressed here as ppbv (10^−9^ = nmol mol^−1^).

For each sample, data from the final 5 min of measurement, corresponding to the steady‐state period, were averaged as the mean ppbv value for each *m/z*. Only *m/z* present in all biological replicates of a given strain were retained for analysis. Emission rates for cultures and controls were calculated following Equation (2):

(2) $${\mathrm{ER}}_{s}=\left(\frac{C\times Q\;}{V}\right)$$

where *ER*_*s*_ is the emission rate of the given BVOC (nmol h^−1^) in the sample; *C* is the volume mixing ratio of a VOC in the sampled air (ppbv); *Q* is the molar flow rate through the jar (mol h^−1^); and *V* is the volume (mL) of the sample.

Final emission rates were calculated following Equation (3):

(3) $${\mathrm{ER}}_{f}=\frac{\left({\mathrm{ER}}_{s1}-{\mathrm{ER}}_{s2}\right)\;}{a}$$

where *ER*_*f*_ is the final emission rate of the given BVOC (nmol cell^−1^ h^−1^); *ER*_*s*1_ is the emission rate of the BVOC in the culture; *ER*_*s*2_ is the emission rate of the BVOC in the control; and *a* is the cell abundance (cells L^−1^). Subtraction of the control was done using a control measurement corresponding to the sample by sampling date.

### 2.5 Phylogenetic Analysis

Nucleotide sequences (Table S1) were retrieved from GenBank (Benson et al. 2018) in R and aligned using the ‘msa’ package with default parameters (Bodenhofer et al. 2015). Pairwise genetic distances were calculated from the alignment using the function ‘dist.alignment’ in ‘seqinr’ package (Charif and Lobry 2007) and a distance‐based phylogeny was then inferred using the BIONJ algorithm implemented in the ‘bionj’ function from the ‘ape’ package (Paradis and Schliep 2019). Pairwise nucleotide identities were subsequently calculated from the same alignment by converting sequences to DNAbin format and computing raw distances with the ‘dist.dna’ function in the ‘ape’ package, which were then transformed to percent identity (1 − distance).

### 2.6 Core Volatilome Analysis

Prevalence and abundance of *m/z* were summarized across replicates by calculating the proportion of strains in which each *m/z* was detected plotted against its total abundance. The common volatilome is defined as *m/z* occurring in ≥ 80% of strains. Abundance thresholds were set for *m/z* occurring in the top 5% and top 1% of total emission rates. From this, those *m/z* in the top 5% of the common volatilome plus the top 1% of the total volatilome were classified as the core volatilome.

### 2.7 Statistical Analysis

All data sets were first assessed for normality using quantile–quantile (*Q*–*Q* plots) and the Shapiro–Wilk test. Due to non‐normality, the overall significance of differences in emission rates among strains and phylum/class were assessed using a Kruskal–Wallis test followed by a post hoc pairwise comparisons performed using a Dunn's test (Dunn 1964) with *p*‐values adjusted with the Benjamini–Hochberg method (Benjamini and Hochberg 1995). The number of *m/z* emitted by each strain was normally distributed and therefore a one‐way analysis of variance (ANOVA) was used to compare strains. The relationship between phylogeny and volatilome was analysed with a principal component analysis (PCA) of emission rates using SIMCA 17 (Sartorius Stedim Data Analytics AB 2021). The distance matrix was based on log‐transformed and unit variance‐scaled data. To assess whether the volatilomes differed among phyla, the extracted principal components (PCs) were analysed using a linear mixed‐effect model (LMM) fitted with maximum likelihood (ML) using the ‘lmer’ function from the lme4 package (Bates et al. 2015), with phylum as a fixed effect and strain as a random intercept. Significance was evaluated using likelihood‐ratio tests comparing models with and without the fixed effect. Differences among strains were assessed using a linear model (LM) followed by a Tukey‐adjusted pairwise comparisons of estimated marginal means (EMM). To assess if the most abundant compounds were responsible for the variation in the score plot, the total and mean squared loadings were calculated for the most abundant *m/z* and the remaining *m/z*. A one‐sided permutation test with 10,000 permutations was used to determine if the mean squared loading of the most abundant *m/z* versus the remaining *m/z* was significantly different.

## 3 Results

The 16 bacterial strains belonged to Alphaproteobacteria, Gammaproteobacteria, Betaproteobacteria, Bacteroidota and Actinomycetes (Figure 1A). Strains showed pair‐wise nucleotide similarities ranging from 64% to 92%, except for isolates BAL228 and BAL217, which shared 100% similarity.

**FIGURE 1:**
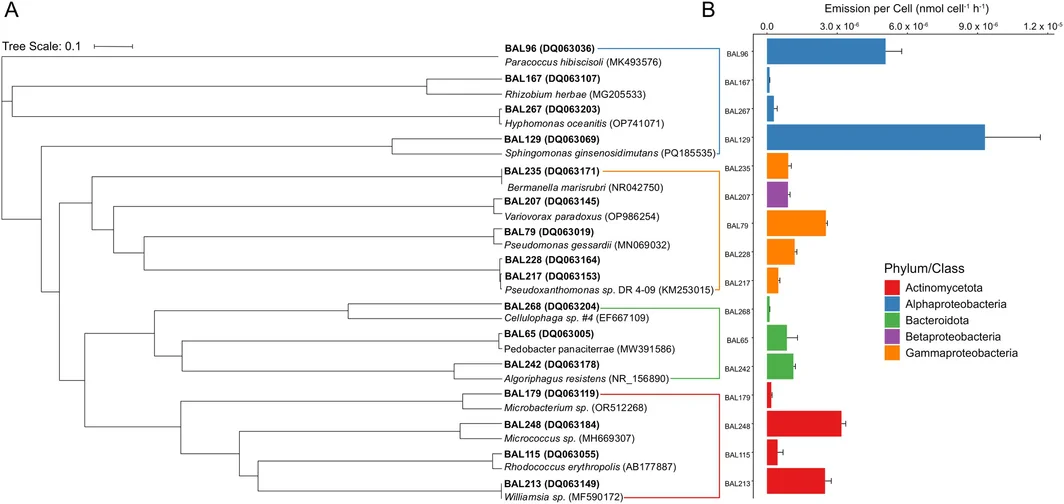
Phylogenetic tree of strains and associated total BVOC emissions. (A) neighbour‐joining phylogenetic tree of the 16 strains (BAL) and the nearest identified neighbour for each (Table S1). The tree scale corresponds to a 10% genetic difference between strains. (B) BVOC emission rate (mean + SE, *n* = 3 except BAL79 = 2) for each strain. Brackets and bars are coloured according to phylum/class.

Quantification of BVOC emissions identified 268 unique *m/z* across all strains. Significant variation in total BVOC emission rates (nmol cell^−1^ h^−1^) was observed among strains (*p* < 0.001; Kruskal–Wallis, Figure 1B). For example, BAL129 exhibited the highest BVOC emission rates (4.7 ± 1.2 × 10^−6^ nmol cell^−1^ h^−1^), followed by BAL96 (2.5 ± 0.4 × 10^−6^ nmol cell^−1^ h^−1^), while strains BAL167 and BAL268 showed the lowest emissions (5.5 ± 0.5 × 10^−8^ and 5.6 ± 0.5 × 10^−8^ nmol cell^−1^ h^−1^, respectively). No significant difference in total emission rates was detected when strains were grouped by their phylum/class (*p* = 0.682; Kruskal–Wallis). However, the small and unequal number of strains per phylum limits statistical interpretation. Identical strains did not exhibit a significant difference in total emission rates (*p* = 0.509; Dunn's test).

Next, we analysed the relative abundances of the 10 most abundant *m/z* for each strain (Figure 2). These comprised 47 discrete *m/z*. Nine were tentatively matched to standards—methanol (*m/z* 33.023), an alkenyl fragment (*m/z* 41.040), acetonitrile (*m/z* 42.013), acetaldehyde (*m/z* 45.035), ethanol (*m/z* 47.051), acetone (*m/z* 59.051), isoprene (*m/z* 69.071), butanone (*m/z* 73.064) and toluene (*m/z* 93.061)—which together with the 39 other tentative compounds (Table S3), accounted for almost 90% of the BVOC profile. Notably, the “Other” fraction in BAL213 accounted for 38.7% (SD = 1.4%). Acetone was the most abundant *m/z* across most strains, comprising between 30.5% and 76.2% of the total abundance, except for BAL129, BAL79, BAL65 and BAL213. BAL129 and BAL79 exhibited the highest proportional contributions from acetaldehyde and ethanol. For BAL129, acetaldehyde accounted for 36.5% (SD = 3.0%) and ethanol 37.2% (SD = 2.2%). BAL65 was characterized by the greatest relative contribution of acetaldehyde at 64.6% (SD = 1.6%). In contrast, BAL213 showed a spread across multiple lower‐abundance *m/z* signals, with *m/z* 87.078 and *m/z* 101.092 accounting for the highest proportional abundances at 12.0% (SD = 9.3%) and 12.0% (SD = 1.2%), respectively. *m/z* 31.012 (formaldehyde), methanol and an alkenyl fragment were also prevalent across strains. Formaldehyde accounted for an average of 3.2% (SD = 3.0%) of relative emissions, methanol for 6.9% (SD = 4.9%) and the alkenyl fragment for 5.9% (SD = 6.7%). The emission rate per hour per cell for formaldehyde, methanol, the alkenyl fragment, acetaldehyde, ethanol, acetone, *m/z* 87.078 and *m/z* 101.092 is presented in Table S4. Notably, the 10 most abundant *m/z* for the phylogenetically identical strains BAL228 and BAL217 demonstrated similar compositions (Figure 2).

**FIGURE 2:**
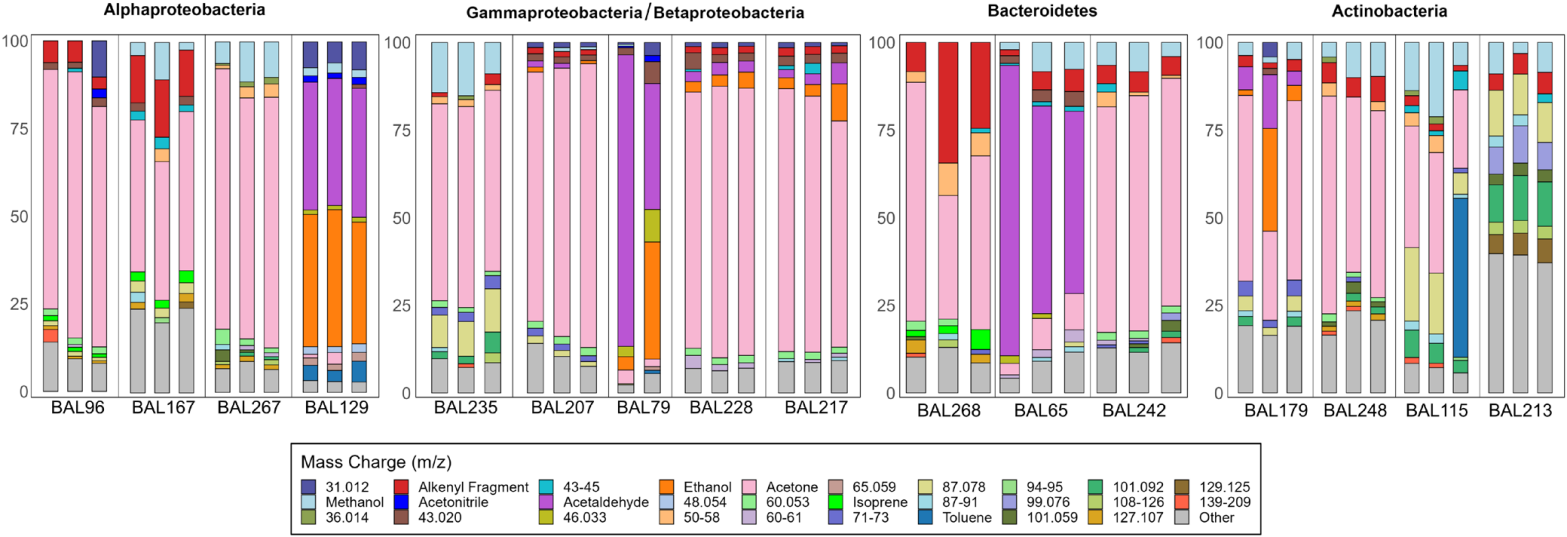
Relative abundance of BVOCs (% of total) for each replicate strain. Strains are grouped by Phylum/Class. The 10 most abundant *m/z* for each strain are included and bars are stacked from top to bottom in *m/z* order, with the grey proportion at the bottom, called ‘Other’, representing the sum of the rest of the masses emitted. *m/z* appear in order from lowest to highest mass. Known *m/z*, tentatively identified using standards, are indicated by their chemical name in the figure legend. Some *m/z* have been grouped for readability (43.057, 44.010 and 45.004; 50.000, 53.005 and 58.073; 60.075 and 61.031; 71.087, 73.038 and 73.064; 87.048, 88.080, 89.057 and 90.953; 94.054 and 95.077; 107.955, 108.954, 115.106, 120.954 and 125.954; 139.075, 153.094, 173.136 and 209.155).

Across the entire volatilome, 26 *m/z* were detected in ≥ 80% of strains and classified as the common volatilome (Figure S1). Common *m/z* varied substantially in their emission rates, ranging from 8.22 × 10^−6^ nmol cell^−1^ h^−1^ for acetone to 1.81 × 10^−6^ nmol cell^−1^ h^−1^ for m/z 176.123. A total of 54% of the *m/z* in the common volatilome were accounted for in the 10 most abundant *m/*z, making up 35.2% of the total emission rate. Only five common *m/z* were present in the top 5% of emissions: acetone, methanol, alkenyl fragment, 87.078 (C_5_H_10_O) and 101.092 (C_6_H_12_O). In addition to these five compounds, acetaldehyde and ethanol were classified as core compounds as their total emission rates were in the 99th percentile.

Between 42 and 182 distinct *m/z* were detected across strains (Table 1). The number of *m/z* produced by each strain was not related to the phylogeny of the strains (*p* > 0.5; ANOVA). Eight strains (BAL96, BAL267, BAL129, BAL207, BAL228, BAL217, BAL248, BAL213) emitted strain‐specific *m/z* (Table 2). These strain‐specific *m/z* were in the lower 0.01% of all emissions, with the most unique *m/z* accounting for less than 0.001% of total emissions. Only the *m/z* 83.085 (C_6_H_10_H^+^) ranked in the top 100 emitted compounds (top 0.03% of all emissions). It accounted for 0.9% of emissions from BAL213 and was the 21st most abundant compound emitted by this strain.

**TABLE 1:** Total number of mass‐to‐charge ratios (*m/z*) and unique *m/z* for each bacterial strain.

	Alphaproteobacteria				Gammaproteobacteria/Betaproteobacteria					Bacteriodota			Actinomycetota			
BAL	96	167	267	129	235	207	79	228	217	268	65	242	179	248	115	213
Total *m/z*	133	116	80	71	53	127	104	182	136	77	107	114	111	134	42	158
Unique *m/z*	2	0	1	12	0	1	0	1	1	0	0	0	0	5	0	4

*Note:* Strains are ordered by phylogeny.

**TABLE 2:** Strain‐specific mass‐to‐charge ratios (*m/z*) and their tentative identification.

Phylum/class	Strain	*m/z*	Tentative ID formula(s); (compound)
Alphaproteobacteria	BAL96	146.107	C_10_H_11_N (3‐Ethyl‐indole); C_7_H_15_NO_2_ (Methyl l‐leucinate)
		194.149	C_12_H_19_NO (e.g., Ethylephedrine or 5,5‐Dimethyl‐3‐pyrrolidino‐cyclohex‐2‐en‐1‐one)
	BAL267	216.215	N/A
	BAL129	66.019	C_4_H_3_N(Cyanoallene)
		103.954	N/A
		117.954	N/A
		118.905	CHCl_3_ (Chloroform)
		118.948	N/A
		131.961	N/A
		137.959	N/A
		147.991	N/A
		161.916	N/A
		164.09	C_9_H_9_NO_2_ (5‐Nitroindan); C_6_H_13_NO_4_ (2‐Nitro‐2‐isopropyl‐1,3‐propanediol); C_10_H_13_NO
		202.16	N/A
		231.97	N/A
Gammaproteobacteria/Betaproteobacteria	BAL207	47.019	CH_2_O_2_ (Formic acid)
	BAL228	186.175	C_12_H_27_N (Tributylamine)
	BAL217	223.082	C_12_H_14_O_4_ (Diethyl Phthalate)
Actinomycetota	BAL248	79.953	N/A
		95.049	C_6_H_6_O (Phenol)
		96.05	^13^C Isotopic Phenol
		192.83	N/A
		194.827	N/A
	BAL213	83.085	C_6_H_10_ (e.g., Cyclohexene, Cis‐1,3‐Hexadiene)
		107.079	C_8_H_10_ (e.g., Ethylbenzene, Xylene)
		125.121	C_9_H_16_ (e.g., 3,3,5‐Trimethylcyclohexene or 1‐Nonyne or 1,3‐Nonadiene)
		141.948	N/A

*Note:* N/A indicates no identification was found. Tentative identification was carried out using the GLOVOCS database (Yáñez‐Serrano et al. 2021) in combination with a targeted literature review.

The relationship between phylogeny and volatilome was analysed using emission rates in a PCA (Figure 3). The first two PCs accounted for 42.0% of the total variance. The strains did not cluster based on phylogeny over PC1 (*p* = 0.636) or PC2 (*p* = 0.740; LMMs on PC scores; Table S5). Both PC1 and PC2 strongly differed by strain (*p* < 0.001; LM on PC scores). Notably, Bacteroidota (BAL65, BAL242, BAL268) and Gammaproteobacteria (BAL79, BAL217, BAL228, BAL235) occupied the centre of the score plot, alongside several Alphaproteobacteria and Actinomycetota strains, suggesting a similar volatilome among these groups. In contrast, BAL129 (Alphaproteobacterium) and BAL213 (Actinomycetota) were divergent on the plot, indicating distinctive volatilomes. The identical strains BAL228 and BAL217 did not differ in their score position across PC1 (*p* = 0.080) or PC2 (*p* = 0.161; Tukey).

**FIGURE 3:**
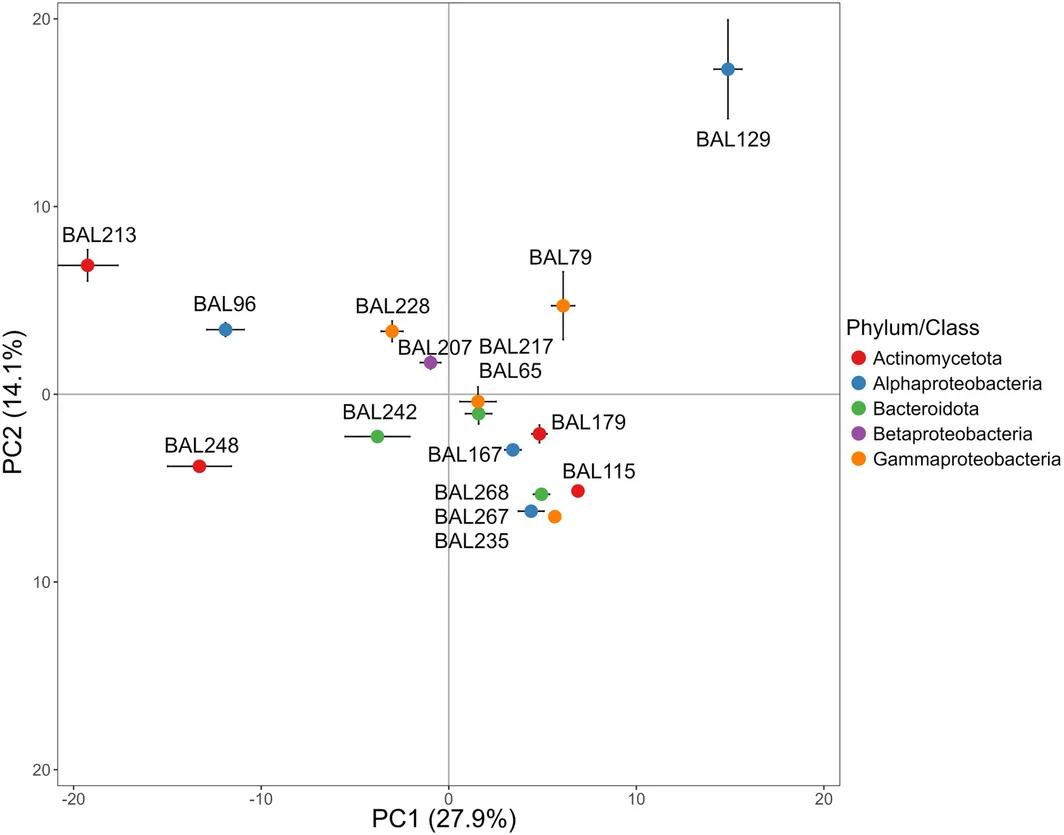
Principal component analysis (PCA) score plot of the total volatilome for the 16 strains (mean PC score position ± SE, *n* = 3 except BAL79 = 2). The volatilomes encompassed 268 unique *m/z*.

The PCA loadings were used to determine whether the most abundant compounds (47 distinct *m/z*; corresponding to coloured fraction in Figure 2) were significant contributors to the strain‐level differences along PC1 and PC2 (Figure S2). The most abundant *m/z* accounted for 19.9% of the total summed squared loadings across PC1 and PC2. In addition, the abundant *m/z* had a slightly higher mean squared loading across PC1 and PC2 than the remaining *m/z* (0.00848 vs. 0.00721), although their contribution was not significant (*p* = 0.075; permutation test).

A pairwise comparison of shared *m/z* among strains revealed a few cases where phylogenetic relatedness and volatilome similarity appear linked (Figure 4). For instance, the identical Gammaproteobacteria BAL228 and BAL217 shared the greatest number of *m/z* (127). Certain strains exhibited a consistently low degree of overlap across the entire panel, regardless of phylogenetic affiliation. For example, BAL129, BAL235 and BAL115 shared relatively few *m/z* features with all other strains. In contrast, BAL213, BAL96, BAL167 and BAL217 shared a relatively high number of features with multiple other strains.

**FIGURE 4:**
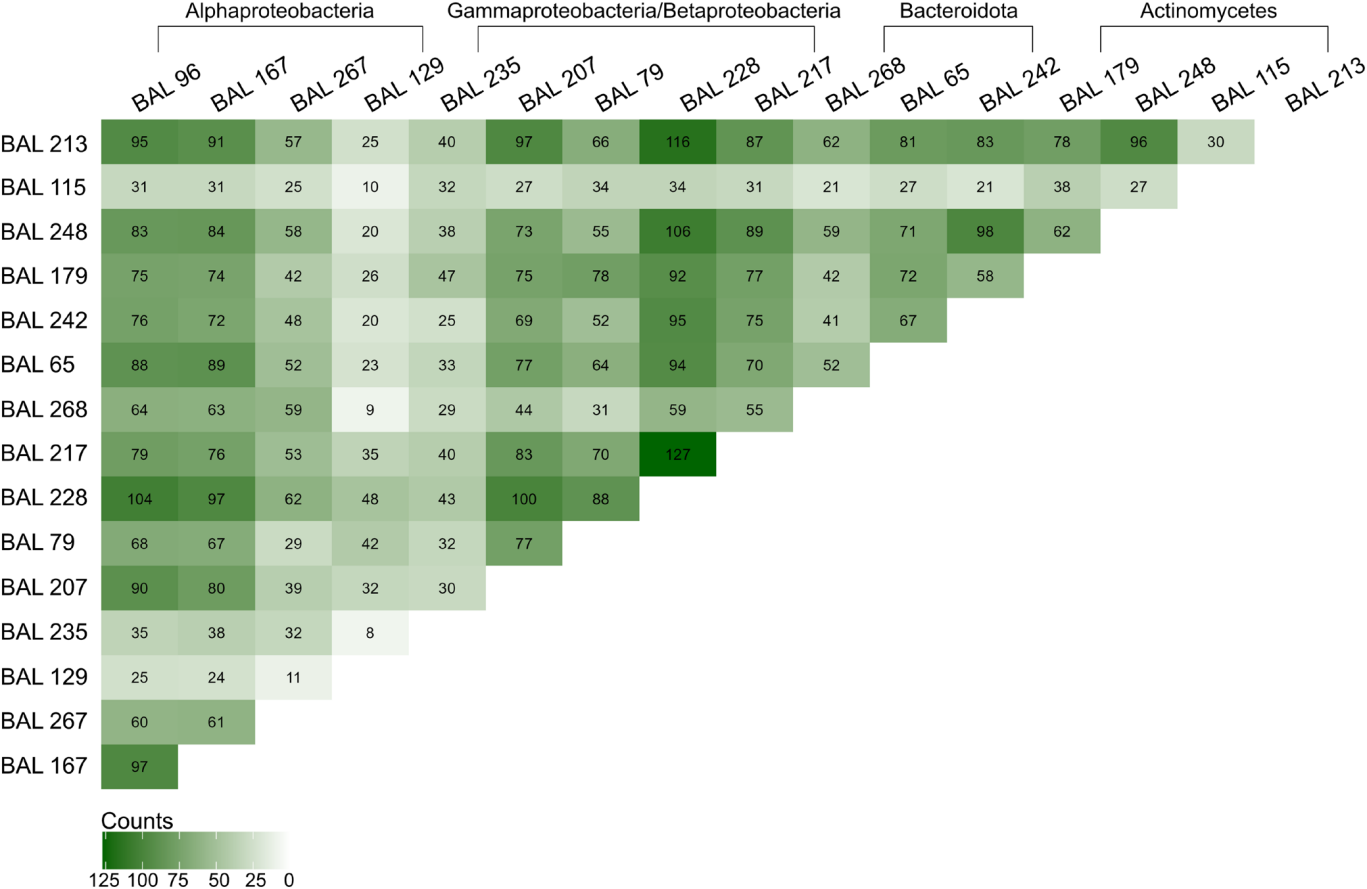
Heatmap showing the total number of mass‐to‐charge ratio (*m/z*) each strain shares with each other. Dark green indicates more shared *m/z*, while light green indicates less shared *m/z*. The number of shared *m/z* is shown inside each rectangle. Strains are ordered by phylogeny.

## 4 Discussion

In this study, we show that 16 phylogenetically diverse Baltic Sea strains emit a rich blend of BVOCs, including a set of core compounds. However, the majority of *m/*z detected were strain‐specific, low‐abundance compounds. The emission patterns did not reflect phylogeny, apart from those strains that shared identity. Overall, this indicates that evolutionary relatedness was not a primary driver of the volatilome structure. Rather, our results suggest the presence of a conserved bacterial volatilome shared across taxa, complemented by a minor subset of strains with distinct emission profiles.

### 4.1 Key Compounds in the Bacterial Volatilome

Across the 16 strains, acetone represented the largest fraction of emissions (50.2%). Similarly, acetone emissions from marine bacteria have been described as early as 1995 by Nemecek‐Marshall et al., who observed substantial production in *Vibrio* isolates from coastal waters off California. Recently, Omori et al. (2025) reported acetone concentrations ~10× higher than MeSH and ~100× higher than acetaldehyde in association with bacterial assemblages from Japanese coastal waters. In both studies, acetone increased with growth and was highest towards the stationary phase, indicating that acetone emissions from the strains measured here could increase in later growth stages. Importantly, acetone production was not universal across the analysed *Vibrio* isolates (Nemecek‐Marshall et al. 1995), consistent with our observation that acetone is a dominant bacterial BVOC but not ubiquitous. Together, the high acetone emission rates observed both in this study and in others suggest that acetone is a key compound emitted by marine bacteria. Moreover, the differences in acetone production between bacterial strains suggest that shifts in bacterial abundance and composition could impact acetone fluxes to the atmosphere. Once in the climate system, acetone can enhance ozone formation and alter the lifetime of methane, acting as a precursor to changes in net radiative forcing (Folkins and Chatfield 2000; Wang et al. 2020). Collectively, these observations suggest that bacterial acetone production should be considered in oceanic acetone cycling models and in the climate system.

Other compounds measured in relatively high abundance included acetaldehyde, ethanol and methanol. These low‐molecular‐weight oxygenated compounds have been widely observed in marine ecosystems (Sinha et al. 2007; Beale et al. 2013; Dixon et al. 2013; Halsey et al. 2017; Davie‐Martin et al. 2020; Zhou et al. 2023) and can originate from bacteria (Kuzma et al. 1995; Bunge et al. 2008; Roslund et al. 2021; Salinas‐García et al. 2026). Yet in the marine environment, few studies have quantified their emission directly from heterotrophic bacteria (Nemecek‐Marshall et al. 1995, 1999; Thiel et al. 2009; Groenhagen et al. 2016; Lawson et al. 2020; Chhalodia et al. 2021), with production commonly attributed to phytoplankton and the role of bacteria commonly described solely in terms of consumption and degradation (Halsey et al. 2017; Wohl et al. 2020; Davie‐Martin et al. 2020; Halsey and Giovannoni 2023). In culture studies, acetaldehyde emissions are commonly associated with early exponential growth (Bunge et al. 2008; Omori et al. 2025; Salinas‐García et al. 2026). This timing fits with our study, indicating that those strains that did not produce acetaldehyde can be considered non‐emitters. For ethanol and methanol, production has been documented in clinically relevant heterotrophic bacteria (Bunge et al. 2008; McNerney et al. 2012; Sovová et al. 2013; Chippendale et al. 2014), but, to our knowledge, no comparable observations exist for marine heterotrophic bacteria. It is important to note that no sulphur compounds, including DMS or MeSH, were tentatively identified in our study despite their ubiquity in the marine environment and known origin from marine bacteria (Brock et al. 2014; Omori et al. 2025). This may reflect both the use of minimal medium and the timing of the measurements. Production of sulphur compounds by heterotrophic bacteria is often linked to the availability of sulphur‐containing precursors, such as amino acids or dimethylsulfoniopropionate (DMSP), which were not present in our minimal medium (Brock et al. 2014; Roslund et al. 2021; Salinas‐García et al. 2026). Likewise, sulphur emissions are often observed during the stationary to death phase when cells have produced complex exudates and cell lysis has deposited organic precursors into the medium for late stage bacteria to utilize (Baumler et al. 2007; Nawrath et al. 2012). Overall, these results highlight that even the emission of the most abundant compounds is highly linked with growth stage and nutrient availability.

### 4.2 A Core Bacterial Volatilome

The analysis identified a broadly overlapping bacterial volatilome during the active growth phase, with compound composition and proportional abundance similar across most strains, irrespective of phylogeny. Gammaproteobacteria, Betaproteobacteria and Bacteroidota had relatively conserved volatilomes, while Actinomycetota and Alphaproteobacteria included strains showing larger variability.

The prevalence analysis (Figure S1) identified a common bacterial volatilome comprising 26 shared *m/z* across ≥ 80% of the strains, providing quantitative evidence of a broadly shared set of BVOCs among phylogenetically diverse marine heterotrophic bacteria. However, most of the common BVOCs were lower‐abundance components of the volatilome, demonstrating a clear distinction between those compounds that are common and those that are highly emitted. This division highlights the need for consideration of both factors when identifying the core volatilome and assessing its potential ecological significance. Therefore, the core volatilome comprised BVOCs that were both common and abundant (acetone, methanol, an alkenyl fragment, C_5_H_10_O, C_6_H_12_O) and acetaldehyde and acetone, which ranked in the top 1% of emissions. Inclusion of the highest abundance compounds in our definition of core compounds is essential for the broader implications of marine bacterial BVOC emissions.

Of the *m/z* observed, between 21 and 116 *m/z* (average 58 *m/z*) were shared across strains of different phylogeny, supporting the presence of a broadly overlapping volatilome (Figure 4). This excludes BAL129, which shared comparatively few (average 24 *m/z*) with the other strains. No single *m/z* was emitted by all strains, although most strains emitted large amounts of acetone (Figure 2). As well, the PCA loadings (Figure S2) were approximately as large for the most abundant and lower‐abundance compounds, showing that both the shared volatilome and the strain‐specific differences accounted for the grouping on the score plot (Figure 3). Overall, the shared bacterial volatilome appears to be dominated by oxygenated BVOCs. Prior work across various ecosystems has observed a similar pattern of emission from heterotrophic bacteria. For example, culture‐based studies have shown that diverse bacterial taxa produce a shared set of low‐molecular‐weight BVOCs despite differences in phylogeny (Bos et al. 2013; Fitzgerald et al. 2021). These commonly shared compounds are often small oxygenated molecules such as alcohols, ketones and aldehydes that arise from conserved pathways of central metabolism (Schulz and Dickschat 2007; Weisskopf et al. 2021). Additionally, carbon substrate composition impacts volatilome composition (Rath et al. 2018; Timm et al. 2018; Jenkins and Bean 2020; Rajendran et al. 2024). Since glucose was the sole carbon source, our emissions likely originate from glycolysis and downstream pathways centered on pyruvate and acetyl‐CoA (Filipiak et al. 2012). Acetone, acetaldehyde, ethanol and other low‐molecular‐weight oxygenated compounds are common end‐products of these pathways (Filipiak et al. 2012; Fitzgerald et al. 2021). This suggests that the overlap observed here reflects emissions of common metabolic end‐products from active growth rather than phylogenetically restricted pathways.

### 4.3 Strain‐Level Differentiation of Volatilomes

The conserved volatilome observed in our study was not universal, as BAL129 (Alphaproteobacteria) and BAL213 (Actinomycetes) clearly diverged from the other strains. BAL129 had the highest emission rate of all strains (Figure 1B), the lowest number of shared *m/z* (Figure 4) and the highest number of unique *m/z* (Table 1). Interestingly, its volatilome was dominated by acetaldehyde and ethanol, with little acetone and no alkenyl fragment production (Figure 2). BAL129 is identical at the 16S rRNA nucleotide level to the soil bacterium *Sphingomonas ginsenosidimutans* (Choi et al. 2010). To our knowledge, BVOC emissions from *S. ginsenosidimutans* have not been examined. Within the *Sphingomonas* genus, BVOC emissions have only been documented from *Pseudomonas* sp. strain 19‐rlim and *Sphingomonas* sp. strain BIR2‐rlima for the terpene 2‐methylisoborneol, an odorous compound sometimes found in drinking water (Eaton 2012). It is unknown whether the high BVOC emission found in BAL129 is a general feature in *Sphingomonas* or specific to our strain. Given that *Sphingomonas* are widely distributed in marine environments (Eguchi et al. 1996; Cavicchioli et al. 1999; Yang et al. 2020) and can dominate coastal estuarine microbial communities during bloom conditions (Piccini et al. 2006), this genus may exert a substantial influence on the volatilome of estuarine systems and, potentially, ecosystem‐scale emissions. A future volatilomic analysis of *Sphingomonas* would be warranted.

BAL213 also differed considerably from the other strains, particularly because its volatilome was dominated by less abundant compounds (Figure 2). In addition, BAL213 did not emit acetone, acetaldehyde or ethanol. Rather, it produced compounds with higher *m/z*, such as 87.078 (C_5_H_10_O), 99.076 (C_6_H_10_O) and 101.092 (C_6_H_12_O). BAL213 is identical at the 16S rRNA nucleotide level to the bacterium *Williamsia* sp. *strain A155*, isolated from Atacama Desert soil (accession MF590172). In a previous study, the strain W*illiamsia* sp. FLCC425 was shown to emit a distinct volatilome and to produce relatively few BVOCs compared to the other Actinobacteria measured (Choudoir et al. 2019). The distinctive volatilome observed for this genus may indicate specialized physiology. In marine waters where low‐abundance taxa contribute outsized functional effects, *Williamsia* could uniquely impact the pools and cycling of low‐abundance reactive gases.

Finally, a set of strains identical in morphology and 16S rRNA nucleotide identity from different stocks were included in our study for the purpose of validating our approach. While the two strains shared more *m/z* than any other pair (127; Figure 4) and exhibited a similar composition of abundant compounds (Figure 2), their volatilomes were not identical (Figure 3) and therefore could not be used for methodological validation. While initially unexpected, our findings are consistent with those of Cordovez et al. (2015), where identical *Streptoyces* isolates emitted different, strain‐specific volatilomes. Volatilome studies may provide finer strain‐level resolution than conventional phylogenetic approaches like 16S rRNA sequencing; however, the high sensitivity of PTR‐ToF‐MS coupled with the dynamic nature of microbial metabolism may lead to the over‐accentuation of differences between otherwise genetically identical strains. Rather, volatilome analysis could be used for indications of community functionality or changing environmental conditions across similar strains.

## 5 Conclusions

Using real‐time PTR‐ToF‐MS volatilomics, we show that phylogenetically diverse heterotrophic bacteria from Baltic Sea surface waters emit chemically complex BVOC blends that include a core volatilome with strain‐specific differentiation. Our findings support a trait‐based view in which strain‐level physiology and substrate availability contribute more strongly to volatilome structure than phylogenetic relatedness (Audrain et al. 2015; Schulz‐Bohm et al. 2017; Meredith and Tfaily 2022). This has implications for how bacterial contributions are represented in marine BVOC budgets, as heterotrophic bacterial emissions are still largely ignored when modelling marine BVOC cycling. Next steps should expand beyond exponential growth and single‐substrate conditions by testing across growth phases, with diverse carbon sources and with mixed communities where interactions can be studied. In addition, future work investigating strain‐specific consumption of BVOCs is a prerequisite for quantifying the net effect of bacteria on BVOC emissions in seawater and elsewhere. Nevertheless, our results demonstrate that heterotrophic marine bacteria emit strain‐specific volatilomes with shared core compounds, supporting the need to incorporate both these abundant compounds and bacterial trait variation into marine microbial models, BVOC budgets and flux estimates.

## Author Contributions

**Eve Galen:** conceptualization, investigation, formal analysis, writing – original draft, writing – review and editing. **Kajsa Roslund:** writing – review and editing, investigation. **Riikka Rinnan:** funding acquisition, writing – review and editing. **Lasse Riemann:** conceptualization, funding acquisition, writing – review and editing.

## Funding

This work was supported by Danmarks Grundforskningsfond (DNRF168).

## Conflicts of Interest

The authors declare no conflicts of interest.

## Supporting information


**Table S1:** Taxonomic identification for the used isolates and their nearest neighbours in GenBank.
**Table S2:** Composition of the gas mixture used for calibration and transmission curves in the Proton Transfer Reaction Time‐of‐Flight Mass Spectrometry.
**Table S3:** Tentative identification of the 10 most abundant mass‐to‐charge ratio (*m/z*) for each strain (38 *m/z* in total). N/A indicates no identification was found. The identification was carried out using the GLOVOCS database (Yáñez‐Serrano et al. 2021) in combination with literature.
**Table S4:** Total BVOC emission rates (nmol cell^−1^ h^−1^ ± SE) of core compounds for each strain. N/A indicates no signal was detected. The *m/z* with associated chemical names were identified using standards.
**Table S5:** Estimated marginal means (EMMs) of principle component scores. EMMs, standard errors (SE), degrees of freedom (df) and 95% confidence internals (CI) are presented for each phylum for PC1 and PC2 from linear mixed‐effects models with phylum as the fixed effect and stain as a random intercept. The overall phylum effect was not significant for PC1 (likelihood‐ratio test, *χ* ^2^ = 2.547, df = 4, *p* = 0.636) or PC2 (*χ* ^2^ = 1.976, df = 4, *p* = 0.740).
**Figure S1:** Prevalence and abundance of total *m/z* for the 16 strains. Prevalence represents the percentage of strains in which each *m/z* was detected. Total abundance represents the total emission rate across strains in which the *m/z* was present. The vertical dashed and dotted line represents 80% and 90% prevalence threshold, respectively. The horizontal dashed and vertical line represent the 95th and 99th percentile, respectively, of total abundance. *m/z* in the common volatilome (≥ 80% prevalence) or *m/z* occurring in the top 5% are labelled. Total abundance is logarithmically scaled. The red box indicates the core volatilome.
**Figure S2:** Loading plot of BVOC emissions for the 16 strains. Red and green points indicate the 47 most abundant *m/z* (corresponding to the coloured fractions in Figure 2), while blue points represent the remaining 221 *m/z*. Green points indicate abundant *m/z* that were also classified as part of the core volatilome. The 47 most abundant *m/z* are labelled.

## Data Availability

The data that supports the findings of this study are available at: https://www.erda.dk/archives/0eb44b6c3d2a5fdac1e549d7694985c3/published‐archive.html.
