# Supplementary material for: Volatile Emissions From Diverse Estuarine Bacteria Share Core Compounds With a Subset of Strain‐Specific, Low Abundance Compounds
Source: Environ Microbiol Rep. 2026 Oct 18;18(5):e70439. doi: 10.1111/1758-2229.70439 (PMC13677163; doi:10.1111/1758-2229.70439)
Supplement: Supplementary file 1 [file EMI4-18-e70439-s001.docx]

**Supporting Information**

**Volatile emissions from diverse estuarine bacteria share core compounds with a subset of strain-specific, low abundance compounds**

Eve Galen^1,2^, Kajsa Roslund^1^, Riikka Rinnan^1^, Lasse Riemann^1,2^

^1^Center for Volatile Interactions, Department of Biology, University of Copenhagen
^2^Marine Biological Section, Department of Biology, University of Copenhagen, Helsingør, Denmark

**Correspondence**: Lasse Riemann (lriemann@bio.ku.dk); Riikka Rinnan (riikkar@bio.ku.dk)

**Table S1.** Taxonomic identification for the used isolates and their nearest neighbors in GenBank.

| **Measured Strains** | | | | | **Nearest Neighbor** | | | | | |
| --- | --- | --- | --- | --- | --- | --- | --- | --- | --- | --- |
| **Name** | **Accession**  **number** | | **Strain** | **Phylum** | **Nearest neighbor** | **Accession**  **number** | **Query cover (%)** | **Percent identity (%)** | **Environment** | **Reference** |
| BAL96 | | DQ063036 | Rhodobacter | Alphaproteobacteria | *Paracoccus hibiscisoli* strain H02Y-003A | MK493576 | 100 | 100.00 | Algal culture | Direct Submission |
| BAL167 | | DQ063107 | Rhizobiaceae | Alphaproteobacteria | Rhizobium herbae | MG205533 | 100 | 99.75 | Soil | Direct Submission |
| BAL267 | | DQ063203 | Hyphomonas | Alphaproteobacteria | Hyphomonas oceanitis | OP741071 | 100 | 100.00 | NA | Direct Submission |
| BAL129 | | DQ063069 | Sphingomonadales | Alphaproteobacteria | Sphingomonas ginsenosidimutans strain HNXS-W42 | PQ185535 | 99 | 100.00 | South China Sea | Direct Submission |
| BAL235 | | DQ063171 | Oceanobacter | Gammaproteobacteria | Bacterium DG940 | NR_042750 | 100 | 100.00 | Red Sea | Pinhassi and Berman 2003 |
| BAL207 | | DQ063145 | Comamonadaceae | Betaproteobacteria | Variovorax paradoxus | OP986254 | 100 | 100.00 | NA | Twizeyimana et al. 2023 |
| BAL79 | | DQ063019 | Pseudomonadaceae | Gammaproteobacteria | Pseudomonas gessardii strain ST3SE | MN069032 | 100 | 100.00 | Soil | Direct Submission |
| BAL228 | | DQ063164 | Xanthomonadaceae | Gammaproteobacteria | Pseudoxanthomonas sp DR4-09 | KM253015 | 100 | 100.00 | Root | Direct Submission |
| BAL217 | | DQ063153 | Xanthomonadaceae 2 | Gammaproteobacteria | Pseudoxanthomonas sp DR4-09 | KM253015 | 100 | 100.00 | Root | Direct Submission |
| BAL268 | | DQ063204 | Flavobacteriaceae | Flavobacteriia | Cellulophaga baltica | EF667109 | 100 | 95.69 | Sea-water | Holmfeldt et al. 2007 |
| BAL65 | | DQ063005 | Sphingobacteriaceae | Sphingobacteriia | Pedobacter panaciterrae strain SCZ9 | MW391586 | 100 | 99.09 | NA | Direct Submission |
| BAL242 | | DQ063178 | Flexibacteriaceae | Cytophagia | Algoriphagus resistens | NR_156890 | 100 | 99.54 | Marine sediment | Han et al. 2017 |
| BAL179 | | DQ063119 | Microbacteriaceae | Actinomycetes | Microbacterium sp. | OR512268 | 100 | 99.75 | Marine sediment | Direct Submission |
| BAL248 | | DQ063184 | Micrococcaceae | Actinomycetes | Micrococcus sp. Strain H390 | MH669307 | 100 | 99.53 | Pine tree | Direct Submission |
| BAL115 | | DQ063055 | Mycobacteriaceae | Actinomycetes | Rhodococcus erythropolis | AB177887 | 100 | 100.00 | NA | Futamata et al. 2004 |
| BAL213 | | DQ063149 | Mycobacteriaceae 2 | Actinomycetes | Williamsia sp. | MF590172 | 100 | 100.00 | Soil | Direct Submission |

**Table S2.** Composition of the gas mixture used for calibration and transmission curves in the Proton Transfer Reaction Time-of-Flight Mass Spectrometry.

| **Compound** | **Molecular formula** | **Molecular mass (u)** |
| --- | --- | --- |
| Acetaldehyde | C_2_H_4_O | 44.05 |
| Methanol | CH_4_O | 32.04 |
| Ethanol | C_2_H_6_O | 46.07 |
| Acetonitrile | C_2_H_3_N | 41.05 |
| Acetone | C_3_H_6_O | 58.08 |
| Isoprene | C_5_H_8_ | 68.12 |
| 2-butanone | C_4_H_8_O | 72.11 |
| Benzene | C_6_H_6_ | 78.11 |
| Toluene | C_7_H_8_ | 92.14 |
| m-Xylene | C_8_H_10_ | 106.16 |
| α-Pinene | C_10_H_16_ | 136.24 |

**Table S3.** Tentative identification of the ten most abundant mass-to-charge ratio (*m/z*) for each strain (38 *m/z* in total). N/A indicates no identification was found. The identification was carried out using the GLOVOCS database (Yáñez-Serrano et al. 2021) in combination with literature.

| ***m/z*** | **Tentative Formula** | **Tentative Compound(s)** |
| --- | --- | --- |
| 31.012 | (CH_2_O)H^+^ | Formaldehyde |
| 36.014 | N/A | N/A |
| 43.02 | (C_2_H_2_O)H^+^ | Acetyl fragment |
| 43.057 | (C_3_H_6_)H^+^ | Propene or alkyl fragment |
| 44.01 | (CHNO)H^+^ | Fulminic acid or isocyanic acid |
| 45.004 | N/A | N/A |
| 46.033 | (CH_3_NO)H^+^ | Formamide |
| 48.054 | (CH_5_NO)H^+^ | o-Methylhydroxylamine or methoxyam |
| 50.003 | N/A | N/A |
| 53.005 | N/A | N/A |
| 58.073 | (C_3_H_7_N)H^+^ | 1-Methylethenylamine or 2-propanimine |
| 60.053 | (C_3_H_7_O)H^+^ | Carbonyl fragment |
| 60.075 | (C_3_H_9_N)H^+^ | Propan-1-amine or propylamine |
| 61.031 | (C_2_H_4_O_2_)H^+^ | Acetic acid |
| 65.059 | (C_2_H_8_O_2_)H^+^ | Ethylene dihydrate |
| 71.087 | (C_5_H_10_)H^+^ | Alkyl fragment (several compounds) |
| 73.038 | (C_3_H_4_O_2_)H^+^ | 2-Propenoic acid or acrylic acid |
| 87.048 | (C_4_H_6_O_2_)H^+^ | Carboxyl-acid derivatives (several compounds) |
| 87.078 | (C_5_H_10_O)H^+^ | Pentanone, pentanal, or 2-methylbutanal |
| 88.08 | (C_4_H_9_NO)H^+^ | N,N-Dimethyl-acetamide |
| 89.057 | (C_4_H_8_O_2_)H^+^ | Ethyl acetate |
| 90.953 | (C_2_H_2_S_2_)H^+^ | 1,2-Dithiete |
| 94.054 | (C_6_H_7_N)H^+^ | Aniline, methylpyridine,  or N-2-oropynyl-2-propyn-1-amine |
| 95.077 | (C_3_H_10_O_3_)H^+^ | Methanol ethylene glycol |
| 99.076 | (C_6_H_10_O)H^+^ | Hexenals (several compounds) |
| 101.059 | (C_5_H_8_O_2_)H^+^ | Acetylacetone |
| 101.092 | (C_6_H_12_O)H^+^ | Hexanal, hexanone, or cis-3-hexenol |
| 107.955 | N/A | N/A |
| 108.954 | (C_2_H_5_Br)H^+^ | Ethyl bromide |
| 115.106 | (C_7_H_14_O)H^+^ | Heptanal, heptanone,  or 2,4-dimethyl-3-pentanone |
| 120.954 | N/A | N/A |
| 125.954 | N/A | N/A |
| 127.107 | (C_8_H_14_O)H^+^ | 6-methyl-5-hepten-2-one (sulcatone)  or 1-Octen-3-one, octenal |
| 129.125 | (C_8_H_16_O)H^+^ | Octanal, octanone, 1-octen-3-ol,  or 2,2,4-trimethyl-3-pentanone |
| 139.075 | (C_8_H_10_O_2_)H^+^ | Creosol |
| 153.094 | (C_9_H_12_O_2_)H^+^ | 4-Ethyl guiacol |
| 173.136 | (C_13_H_16_)H^+^ | 3-heptynylbenzene |
| 209.155 | (C_13_H_20_O_2_)H^+^ | Carvyl propionate |

**Table S4.** Total BVOC emission rates (nmol cell^-1^ h^-1^ ± SE) of core compounds for each strain. N/A indicates no signal was detected. The *m/z* with associated chemical names were identified using standards.

| **Strain** | **Methanol**  **(33.023)** | **Alkenyl Fragment**  **(41.04)** | **Acetaldehyde**  **(45.035)** | **Ethanol**  **(47.051)** | **Acetone (59.051)** | **87.078** | **101.092** |
| --- | --- | --- | --- | --- | --- | --- | --- |
| BAL96 | N/A | 1.04 ± 0.21 x 10^-7^ | N/A | N/A | 1.39 ± 0.66 x 10^-6^ | 2.44 ± 0.41 x 10^-8^ | 1.17 ± 0.32 x 10^-8^ |
| BAL167 | 9.15 ± 3.32 x 10^-10^ | 2.56 ± 0.40 x 10^-9^ | N/A | N/A | 7.77 ± 1.56 x 10^-9^ | 5.50 ± 1.25 x 10^-10^ | 2.43 ± 0.89 x 10^-10^ |
| BAL267 | 2.06 ± 0.45 x 10^-9^ | N/A | N/A | N/A | 1.79 ± 0.68 x 10^-8^ | N/A | 1.62 ± 0.36 x 10^-10^ |
| BAL129 | 1.09 ± 0.22 x 10^-7^ | N/A | 1.70 ± 0.44 x 10^-6^ | 1.72 ± 0.43 x 10^-6^ | 5.14 ± 2.23 x 10^-8^ | N/A | N/A |
| BAL235 | 1.18 ± 0.17 x 10^-8^ | 1.62 ± 0.83 x 10^-9^ | N/A | N/A | 5.15 ± 0.44 x 10^-8^ | 9.99 ± 1.76 x 10^-9^ | 3.33 ± 1.50 x 10^-9^ |
| BAL207 | 3.59 ± 0.52 x 10^-9^ | 5.78 ± 0.21 x 10^-9^ | 4.50 ± 1.01 x 10^-9^ | 4.10 ± 0.43 x 10^-9^ | 2.75 ± 0.27 x 10^-7^ | 6.09 ± 0.64 x 10^-9^ | 3.32 ± 0.63 x 10^-9^ |
| BAL79 | 6.14 ± 0.16 x 10^-9^ | 2.88 ± 0.56 x 10^-9^ | 7.34 ± 2.79 x 10^-7^ | 2.34 ± 1.88 x 10^-7^ | 3.72 ± 1.02 x 10^-8^ | 3.19 ± 0.60 x 10^-9^ | 1.32 ± 0.22 x 10^-9^ |
| BAL228 | 7.70 ± 2.11 x 10^-10^ | 9.89 ± 1.18 x 10^-9^ | 1.67 ± 0.20 x 10^-8^ | 1.91 ± 0.46 x 10^-8^ | 3.92 ± 0.42 x 10^-7^ | 1.62 ± 0.15 x 10^-9^ | 1.20 ± 0.17 x 10^-9^ |
| BAL217 | N/A | 3.95 ± 1.18 x 10^-9^ | 8.08 ± 4.83 x 10^-9^ | 1.30 ± 9.16 x 10^-9^ | 1.23 ± 0.34 x 10^-7^ | 1.16 ± 0.51 x 10^-9^ | 4.77 ± 2.40 x 10^-10^ |
| BAL268 | N/A | 2.47 ± 0.71 x 10^-9^ | N/A | N/A | 6.96 ± 2.49 x 10^-9^ | N/A | N/A |
| BAL65 | 1.13 ± 0.19 x 10^-8^ | 8.79 ± 2.02 x 10^-9^ | 2.43 ± 1.77 x 10^-7^ | N/A | 1.56 ± 0.42 x 10^-8^ | 1.84 ± 0.49 x 10^-9^ | 7.96 ± 2.75 x 10^-10^ |
| BAL242 | 8.10 ± 1.22 x 10^-9^ | 8.02 ± 2.79 x 10^-9^ | N/A | N/A | 9.65 ± 3.45 x 10^-8^ | 1.05 ± 0.58 x 10^-9^ | 2.18 ± 1.20 x 10^-9^ |
| BAL179 | 1.85 ± 0.23 x 10^-9^ | 1.52 ± 0.24 x 10-9 | 6.55 ± 3.91 x 10^-9^ | 9.87 ± 8.63 x 10^-9^ | 2.45 ± 0.42 x 10^-8^ | 2.02 ± 0.30 x 10^-9^ | 1.30 ± 0.20 x 10^-9^ |
| BAL248 | 3.36 ± 1.23 x 10^-8^ | 2.41 ± 0.66 x 10^-8^ | N/A | N/A | 2.06 ± 0.37 x 10^-7^ | 3.38 ± 1.35 x 10^-9^ | 7.08 ± 2.63 x 10^-9^ |
| BAL115 | 2.25 ± 0.42 x 10^-8^ | 4.18 ± 1.58 x 10^-9^ | N/A | N/A | 5.94 ± 2.20 x 10^-8^ | 2.33 ± 0.30 x 10^-8^ | 1.04 ± 0.32 x 10^-8^ |
| BAL213 | 2.50 ± 1.07 x 10^-8^ | 1.87 ± 0.63 x 10^-8^ | N/A | N/A | N/A | 3.93 ± 1.00 x 10^-8^ | 4.02 ± 1.23 x 10^-8^ |

**Table S5.** Estimated marginal means (EMMs) of principle component scores. EMMs, standard errors (SE), degrees of freedom (df), and 95% confidence internals (CI) are presented for each phylum for PC1 and PC2 from linear mixed-effects models with phylum as the fixed effect and stain as a random intercept. The overall phylum effect was not significant for PC1 (likelihood-ratio test, χ² = 2.547, df = 4, p = 0.636) or PC2 (χ² = 1.976, df = 4, p=0.740).

| **PC** | **Phylum** | **EMM** | **SE** | **df** | **CI low** | **CI high** |
| --- | --- | --- | --- | --- | --- | --- |
| PC1 | Actinobacteria | -5.21 | 4.69 | 23.26 | -14.90 | 4.49 |
| PC1 | Alphaproteobacteria | 2.70 | 4.69 | 23.26 | -7.00 | 12.40 |
| PC1 | Bacteroidetes | 0.91 | 5.42 | 23.26 | -10.29 | 12.11 |
| PC1 | Betaproteobacteria | -0.97 | 9.38 | 23.26 | -20.37 | 18.43 |
| PC1 | Gammaproteobacteria | 2.57 | 4.69 | 23.29 | -7.14 | 12.27 |
| PC2 | Actinobacteria | -1.06 | 3.37 | 23.25 | -8.03 | 5.91 |
| PC2 | Alphaproteobacteria | 2.89 | 3.37 | 23.25 | -4.08 | 9.86 |
| PC2 | Bacteroidetes | -2.87 | 3.89 | 23.25 | -10.92 | 5.17 |
| PC2 | Betaproteobacteria | 1.69 | 6.74 | 23.25 | -12.25 | 15.62 |
| PC2 | Gammaproteobacteria | 0.28 | 3.37 | 23.30 | -6.70 | 7.25 |

**Figure S1.** Prevalence and abundance of total *m/z* for the 16 strains. Prevalence represents the percentage of strains in which each *m/z* was detected. Total abundance represents the total emission rate across strains in which the *m/z* was present. The vertical dashed and dotted line represents 80% and 90% prevalence threshold, respectively. The horizontal dashed and vertical line represent the 95^th^ and 99^th^ percentile, respectively, of total abundance. *m/z* in the common volatilome (≥80% prevalence) or *m/z* occurring in the top 5% are labeled. Total abundance is logarithmically scaled. The red box indicates the core volatilome.

**Figure S2.** Loading plot of BVOC emissions for the 16 strains. Red and green points indicate the 47 most abundant *m/z* (corresponding to the colored fractions in Figure 2), while blue points represent the remaining 221 *m/z*. Green points indicate abundant *m/z* that were also classified as part of the core volatilome. The 47 most abundant *m/z* are labelled.

**References**

Futamata H, Uchida T, Yoshida N, et al (2004) Distribution of Dibenzofuran-Degrading Bacteria in Soils Polluted with Different Levels of Polychlorinated Dioxins. Microb Environ 19:172–177. https://doi.org/10.1264/jsme2.19.172

Han J-R, Zhao J-X, Wang Z-J, et al (2017) Algoriphagus resistens sp. nov., isolated from marine sediment. International Journal of Systematic and Evolutionary Microbiology 67:1275–1280. https://doi.org/10.1099/ijsem.0.001805

Holmfeldt K, Middelboe M, Nybroe O, Riemann L (2007) Large Variabilities in Host Strain Susceptibility and Phage Host Range Govern Interactions between Lytic Marine Phages and Their Flavobacterium Hosts. Applied and Environmental Microbiology 73:6730–6739. https://doi.org/10.1128/AEM.01399-07

Pinhassi J, Berman T (2003) Differential Growth Response of Colony-Forming α- and γ-Proteobacteria in Dilution Culture and Nutrient Addition Experiments from Lake Kinneret (Israel), the Eastern Mediterranean Sea, and the Gulf of Eilat. Applied and Environmental Microbiology. https://doi.org/10.1128/AEM.69.1.199-211.2003

Twizeyimana M, Hammer PE, Gachango E, et al (2023) Diverse environmental bacteria displaying activity against Phakopsora pachyrhizi, the cause of soybean rust. Front Plant Sci 14:. https://doi.org/10.3389/fpls.2023.1080116

Yáñez-Serrano AM, Filella I, LLusià J, et al (2021) GLOVOCS - Master compound assignment guide for proton transfer reaction mass spectrometry users. Atmospheric Environment 244:117929. https://doi.org/10.1016/j.atmosenv.2020.117929
